# Softness enhanced macrophage-mediated therapy of inhaled apoptotic-cell-inspired nanosystems for acute lung injury

**DOI:** 10.1186/s12951-023-01930-2

**Published:** 2023-05-30

**Authors:** Dazheng Sun, Guanglin Zhang, Mingyang Xie, Yina Wang, Xiangchao Liang, Mei Tu, Zhijian Su, Rong Zeng

**Affiliations:** 1 Department of Materials Science and Engineering, College of Chemistry and Materials, Jinan University, Guangzhou, 510632 P. R. China; 2 Henry Fok Colloge of Biology and Agriculture, Shaoguan University, Shaoguan, 512005 P. R. China; 3 Guangdong Provincial Key Laboratory of Bioengineering Medicine, Department of Cell Biology, Jinan University, Guangzhou, 510632 P. R. China; 4 National Engineering Research Center of Genetic Medicine, Jinan University, Guangzhou, 510632 P. R. China

**Keywords:** Softness, Apoptotic-cell-inspired, Macrophage, Anti-inflammatory, Acute lung injury

## Abstract

Engineered nanosystems offer a promising strategy for macrophage-targeted therapies for various diseases, and their physicochemical parameters including surface-active ligands, size and shape are widely investigated for improving their therapeutic efficacy. However, little is known about the synergistic effect of elasticity and surface-active ligands. Here, two kinds of anti-inflammatory *N*-acetylcysteine (NAC)-loaded macrophage-targeting apoptotic-cell-inspired phosphatidylserine (PS)-containing nano-liposomes (PSLipos) were constructed, which had similar size and morphology but different Young’s modulus (E) (H, ~ 100 kPa > E_macrophage_ vs. L, ~ 2 kPa < E_macrophage_). Interestingly, these PSLipos-NAC showed similar drug loading and encapsulation efficiency, and in vitro slow-release behavior of NAC, but modulus-dependent interactions with macrophages. Softer PSLipos-L-NAC could resist macrophage capture, but remarkably prolong their targeting effect period on macrophages via durable binding to macrophage surface, and subsequently more effectively suppress inflammatory response in macrophages and then hasten inflammatory lung epithelial cell wound healing. Especially, pulmonary administration of PSLipos-L-NAC could significantly reduce the inflammatory response of M1-like macrophages in lung tissue and promote lung injury repair in a bleomycin-induced acute lung injury (ALI) mouse model, providing a potential therapeutic approach for ALI. The results strongly suggest that softness may enhance ligand-directed macrophage-mediated therapeutic efficacy of nanosystems, which will shed new light on the design of engineered nanotherapeutics.

**Graphical abstract:**

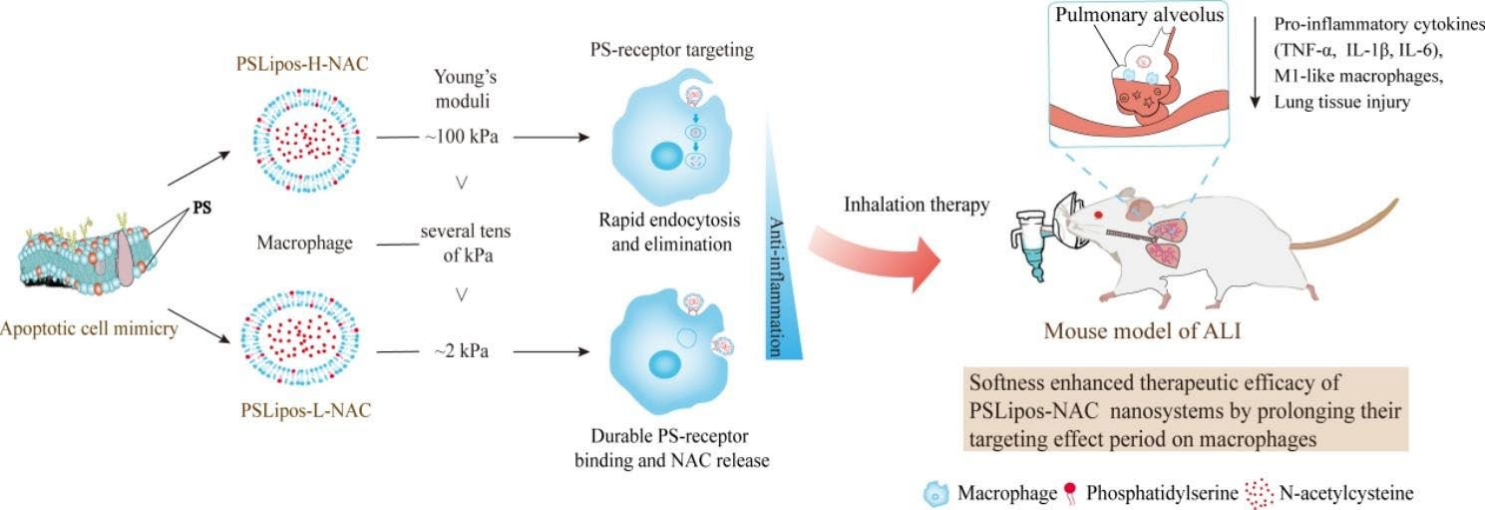

**Supplementary Information:**

The online version contains supplementary material available at 10.1186/s12951-023-01930-2.

## Introduction

Macrophage-based therapies have emerged as prospective therapeutic strategies for various cancers, acute and chronic inflammatory diseases, infection and regeneration medicine due to the fact that macrophages play the crucial role in development, homeostasis, tissue repair, and immunity [1–4]. Engineered nanoparticles have attracted great attentions in macrophage-targeted drug delivery systems for achieving desired therapeutic effects, attributing to their unique physicochemical and biological properties for not only effectively loading therapeutic agents but also on-demand regulating macrophage responses. Recent studies have claimed that the physicochemical parameters of engineered nanoparticle-based carriers (e.g., composition, size, shape, charge, surface properties and elasticity) can be altered to direct their interactions with macrophages for better outcome [5–7]. Surface functionalization of nanoparticles with receptor-targeting ligand moieties is generally considered as a powerful approach to activate macrophages and increase drug uptake by macrophages via the ligand-receptor interactions. Meanwhile, softening the nanoparticles has proven to be beneficial in avoiding macrophage phagocytosis, hence resulting in their prolonged half-life in circulation [8] and better accumulation in tumor region. However, it remains largely unknown how nanoparticle elasticity impacts on the therapeutic performance of the ligand-directed active macrophage-targeting nanoparticle-based drug delivery systems.

Apoptotic cells with phosphatidylserine (PS) signal exposed on the surface can be recognized and eliminated by macrophages, and subsequently activate anti-inflammatory and immunoregulatory responses of macrophages, therefore apoptotic-cell-inspired PS-containing liposomes provided an attractive macrophage-targeting platform for treating various inflammatory diseases and injuries, such as myocardial infarction, rheumatoid arthritis, and retinal ischemia-reperfusion injury [9–12]. Our previous work showed that lower Young’s modulus (E) of PS-containing deformable nano-liposomes would greatly affect PS-receptor-mediated macrophage responses, including cellular internalization, anti-inflammatory and pro-healing cytokine production, but the synergistic effects of drug-loaded PS-containing deformable nano-liposomes on macrophage-based therapy for inflammatory injuries remain much less understood [13]. Actually, although deformable liposomes containing edge activators with lower modulus are routinely employed for more efficient transdermal delivery of therapeutically active molecules via intercellular pathways due to their excellent flexibility and deformability, the study of how they interact with macrophages is commonly ignored [14].

Acute lung injury (ALI) and its most severe manifestation, acute respiratory distress syndrome (ARDS), represent a critical clinical syndrome with high rate of morbidity and mortality caused by various direct and indirect injury factors, leading to the inflammatory damage of alveolar epithelium and pulmonary endothelial cells, and place a tremendous burden on the healthcare system worldwide [15, 16]. Coronavirus Disease 2019 (Covid-19) caused by severe acute respiratory syndrome coronavirus-2 (SARS-CoV-2) most commonly manifests with ALI/ARDS, exhibiting substantial high mortality [17–19]. According to clinical evidence, inflammatory cells and mediators obviously promote and exacerbate lung inflammation, and macrophages in particular are important in the overall pathophysiology of ALI/ARDS [20, 21]. In the acute phase of ALI/ARDS, lung macrophages exhibit a predominant pro-inflammatory phenotype (M1), releasing a variety of potent pro-inflammatory and deleterious mediators such as tumor necrosis factor α (TNF-α), interleukin 1 (IL-1), nitric oxide (NO), and reactive oxygen species (ROS), all of which can cause an uncontrolled inflammatory response and excessive oxidative stress to damage the lung tissue. In this study, apoptotic-cell-inspired PS-containing nano-liposomes (PSLipos) with similar size but different elasticity were used to load *N*-acetylcysteine (NAC) as an inhalant for macrophage-based therapy of ALI. NAC is a known antioxidant and anti-inflammatory agent with a strong ability to scavenge ROS and inhibit the expression of inflammatory cytokines in macrophages, and widely used in the management of some respiratory diseases, including ALI/ARDS, bronchitis and asthma [4, 19, 21]. Based on the modulus of macrophages reported by Souza et al. to be approximately several tens of kPa [22], we investigated the construction of PSLipos-NAC nanotherapeutic systems with two different modulus (H, ~ 100 kPa > E_macrophage_ vs. L, ~ 2 kPa < E_macrophage_), and their modulus-dependent synergistic effects on macrophages, as well as their therapeutic effects on a bleomycin-induced ALI mouse model via pulmonary administration. We anticipate that this study will benefit the development of engineered nanoparticle-based platforms for immunotherapy, as well as give a potential therapeutic option for ALI.

## Experimental section

### Materials

*N*-acetylcysteine (NAC) was obtained from Shanghai Aladdin Biochemical Technology Co., Ltd. (China). Soybean phosphatidylcholine (SPC), cholesterol (Chol), sodium deoxycholate (SDC) and lipopolysaccharides (LPS) were purchased from Shanghai Macklin Biochemical Co., Ltd. (China). 1, 2-Dipalmitoyl-sn-glycero-3-phospho-L-serine (DPPS) was available from Corden Pharma Switzerland LLC. Dulbecco’s modified Eagle medium (DMEM), fetal bovine serum (FBS), L-glutamine, sodium pyruvate, PBS buffer and penicillin-streptomycin (P/S) were obtained from ThermoFisher Scientific Inc. (USA). All other solvents were of analytical grade or specific HPLC grade. Recombinant murine M-CSF was purchased from PeproTech, Inc. (USA). Bleomycin (BLM) was obtained from Hanhui Pharmaceuticals Co., Ltd. (China).

### Cell lines and animals

Bone marrow derived macrophages (BMDMs) were harvested and differentiated from 6-week-old C57BL/6 mice [23]. Briefly, bone marrow was flushed from mouse femurs with cold PBS. After filtering using cell strainers of 100 μm (BD Biosciences), cells were harvested by centrifugation at 800 rpm for 10 min and then red blood cells were lysed for 10 min on ice. Cells were cultured for 7 days in 6 cm Petri dishes using DMEM medium containing 10% FBS, P/S (100 µg/mL) and M-CSF (50 ng/mL) at a density of 10^6^ cells/mL. The obtained BMDMs were used as M0 macrophages. M1 macrophages were obtained by further 12 h incubation with 500 ng/mL LPS [24]. The RAW264.7 cells and mouse lung epithelial 12 (MLE‑12) cells obtained from ATCC were incubated in complete medium (DMEM with 10% FBS) at 37 °C and 5% CO_2_.

Male BALB/C and C57BL/6 mice (6–8 weeks of age) were purchased from Guangdong Medical Laboratory Animal Center (Guangdong, China) and housed at the Animal Center of Jinan University. All experimental procedures involving animals were approved by the Jinan University Laboratory Animal Ethics Committee. Maximum care was taken to limit the number of animals used in this study. Mice were anesthetized with 1% sodium pentobarbital (45 mg/kg) via intraperitoneal injection to ensure free of pain for any invasive operations.

### Preparation and characterization of PSLipos and PSLipos-NAC

Two kinds of apoptotic-cell-inspired PS-containing nano-liposomes with low and high modulus (PSLipos-L and -H) were prepared through the combined method of the thin-film hydration, ultrasonication and extrusion [25]. Briefly, SPC, DPPS and Chol were dissolved in chloroform/ethanol solution (5:1, v/v) in round-bottomed flasks with molar ratios of 6.5:1.5:2. Then organic solvents were removed using rotary evaporator at 37 °C. For preparing PSLipos-L or -H, the lipid film was then hydrated in the SDC solutions (PBS buffer, pH 7.4, with the molar ratio of Chol to SDC = 1:0.4) or PBS buffer (pH 7.4) by vigorous shaking and vortex mixing for 30 min at 52 °C. The total lipid concentration was 10 mM. The resulting liposomes were probe-sonicated (150 W) for 3 min under ice bath condition, then homogenized by successive extrusion through a 100 nm pore size polycarbonate membrane (Nucleopore®, Whatman Inc) at 52 °C for 3 times.

The loading of NAC into PSLipos was performed by pH gradient method [26]. Briefly, SPC, DPPS and Chol were dissolved in chloroform/ethanol solution (5:1, v/v) in round-bottomed flasks with molar ratios of 6.5:1.5:2, and then evaporated in a water bath at 37 °C under reduced pressure until the formation of even thin film was observed. Thereafter, SDC/NaHCO_3_-NaOH buffer solution (pH 10.4) with the molar ratio of Chol to SDC = 1:0.4 was added, and the mixture was incubated again in a water bath at 52 °C for 30 min, sonicated and homogenized through a 100 nm polycarbonate membrane to obtain the pre-formed blank liposomes (pH 10.4), which were further dialyzed in FLOAT-A-LYZER G2 dialysis tubing (MWCO 10 kDa, Spectrum) in PBS (pH 7.4) overnight at 4 °C to adjust the external pH of liposomes to certain pH 7.4. Then, NAC solution (10 mg/mL) was added into the liposome dispersion and incubated for 2 h at 52 °C to obtain the PSLipos-L-NAC with 10 mM of the final total lipid concentration. And all drug-loaded liposomes were purified by the ultrafiltration/centrifugation technique (MWCO 10 kDa, Millipore) at 3000 *g* for 10 min. PSLipos-H-NAC was made using the similar procedure without the addition of SDC.

The particle size, polydispersity index (PDI) and Zeta potential of liposomes were determined by Zetasizer (Nano-ZS, Malvern Instruments, Malvern, UK). The encapsulation efficiency (EE) and drug loading efficiency (DLE) of NAC were calculated by equations: EE% = (the mass of total fed NAC - the mass of unentrapped NAC) / (the mass of total fed NAC) × 100%, and DLE% = (the mass of total fed NAC - the mass of unentrapped NAC) / (the mass of total NAC-loaded liposomes) × 100%, respectively. The content of NAC was measured by UV-Vis spectrophotometry (Persee TU-1810SPC, China) at 207 nm [27]. The morphology of the liposomes was observed by transmission electron microscopy (TEM) [28]. Briefly, one drop of the liposome dispersion was placed onto a holey formvar-coated copper grid (230 mesh, round fields). The excess liquid was sucked away by filter paper. After stained with 2% sodium phosphotungstate solution at room temperature for 2 min and air-dried, the TEM images were obtained on a JEM-1400flash microscopy (JEOL, Japan). The Young’s modulus of liposomes was determined using an atomic force microscope (AFM, Bruker Dimension FastScan) based on the Hertz model according to our previous work [13].

### In vitro drug release and stability of PSLipos-NAC

In order to investigate the drug release behavior of liposomal formulations in the medium similar to the extracellular environment of the lung, the Gamble’s solution (its chemical composition was shown in Table S2) which simulates the interstitial lung fluid found within the deep lung was used as the drug release medium [29–31]. The in vitro release of NAC from PSLipos-L-NAC or PSLipos-H-NAC was performed using dialysis method. The free NAC solution and PSLipos-NAC dispersion were individually placed into a dialysis tubing (MWCO 10 kDa). The dialysis devices were separately sunk in Gamble’s solution (pH 7.4) at 37 °C under stirring at 100 rpm. At indicated time points, the 2 mL of the release media was collected and equal volume fresh media was added. The NAC concentration in the collected samples was determined by UV-Vis spectrophotometry (Persee TU-1810SPC, China) at 207 nm.

Additionally, we also performed the long-term and accelerated physical stability testing on the liposomes [26, 32]. The fresh as-prepared liposomal suspensions were subjected to centrifugation (3200 *g* for 1 h at 25 °C) or horizontal mechanical stirring (180 beats/min for 48 h at 37 °C) for accelerated testing, and stored at 4 °C for 30 days for long-term stability testing. The macroscopic appearance, pH change, particle size and PDI, as well as zeta potential of the liposomes were monitored.

### In vitro cytotoxicity, anti-inflammatory and pro-healing assays

#### Cytotoxicity evaluation

The cytotoxicity of samples with respect to BMDMs and MLE‑12 cells was measured by Cell Counting Kit-8 (CCK-8, KeyGEN, China). The BMDMs and MLE‑12 cells were seeded into 96-well cell culture plates at the density of 5000 and 3000 cells per well, respectively. After 24 h cultivation, the cells were incubated with different concentrations of free NAC, blank PSLipos and PSLipos-NAC with different modulus for 48 h. The relative cell viability was determined through the CCK-8 assay with following formula Eq. (1):

1 $$Viability=\frac{{OD}_{\text{Sample}}\text{-}{OD}_{Base}}{{OD}_{C\text{ontrol}}\text{-}{OD}_{Base}}\times 100\text{\%}$$

#### Macrophage capture and intracellular distribution

Overall, 10^4^ RAW264.7 cells were seeded in each well of a 96-well plate and allowed to attach overnight. The cells were incubated with 0.5 µmol/mL total lipids of fluorescein-DHPE-labeled PSLipos (molar ratio, fluorescein-DHPE: total lipids = 6:1000) in a DMEM medium for 24 h. Next, the cells were washed with PBS three times and the fluorescence was quantified using a Flow Cytometer (BD FACSCanto). The mean fluorescence intensity (MFI) calculated from at least three independent experiments was used to determine macrophage capture efficiency.

To study the intracellular distribution of PSLipos, the cells were pretreated with 0.5 µmol/mL total lipids of fluorescein-DHPE-labeled PSLipos for 3 h in the DMEM medium, replaced with a fresh medium and cultivated for 0 to 24 h. Next, the cells were washed with PBS three times and the fluorescence was quantified using a microplate reader (Cytation3, BioTek). The images at 3 and 12 h was further investigated using a confocal fluorescence microscope. Briefly, the cells were washed with PBS three times and fixed with 4% paraformaldehyde for 15 min. Cell membrane were labeled for 20 min at 25 °C with 10 µM DIL (Invitrogen). Then, the cells were observed under a confocal fluorescence microscope (LSM 880 with AiryScan, Carl Zeiss).

#### Detection of intra- and extracellular concentrations

10^6^ RAW264.7 cells were seeded in each well of a 6-well plate and induced by 500 ng/mL LPS for 12 h to obtain the M1 phenotype. The cells were pretreated with 61.25 µg/mL NAC and PSLipos-NAC dispersion (0.5 µmol/mL of the total lipid concentration, containing 61.25 µg/mL NAC) for 3 h in the DMEM medium, then replaced with a fresh medium and cultivated for 0 to 24 h. Next, the cell supernatant was collected to determine extracellular NAC concentrations using a NAC ELISA kit (Meimian, Jiangsu, China), and the cells were also collected and lysed to determine intracellular NAC concentrations. To exclude interference from endogenous NAC in experiments as much as possible, background correction was performed for all the measurements to obtain the exogenous extra- and intra-cellular NAC concentrations due to free NAC or PSLipos-NAC treatment by subtracting the endogenous NAC in M1 macrophages at the corresponding time point.

#### Real‑time quantitative PCR

BMDMs were seeded on 6-well plates (7 × 10^5^ cells/well) and cultured for 12 h, then induced by 500 ng/mL LPS for another 12 h to obtain the M1 polarization state, whereas the cells untreated with LPS were considered as the control group. The M1 macrophages were then treated with 122.5 µg/mL NAC, PSLipos (1mM of the total lipid concentration) and PSLipos-NAC dispersion (1mM of the total lipid concentration, containing 122.5 µg/mL NAC) for 24 h, respectively. Next, the cells were washed with PBS, and the total RNA was isolated and purified from the cultured cells using HiPure Total RNA Mini Kit according to the manufacturer’s instruction. cDNA was prepared using HiScript® II Q RT SuperMix for qPCR (+ gDNA wiper) (Vazyme, China). Real-time qPCR experiments were performed using AceQ® qPCR SYBR® Green Master Mix (Vazyme, China) in a CFX96 Real-Time PCR Detection System (Bio-Rad). Glyceraldehyde 3-phosphate dehydrogenase (GAPDH) was used as an endogenous reference gene to normalize the results. Relative gene expressions were calculated by ΔΔCt method relative to the control group. The primer sequences used in the experiments were as follows:

TNF-α: 5'-GGCAGGTCTACTTTGGAGTCATTGC-3' and 5’-ACATTCGAGGCTCCAGTGAATTCGG-3'; IL-1β: 5'-TGCCACCTTTTGACAGTGATG − 3' and 5’-TGATACTGCCTGCCTGAAGC-3'; IL-6: 5'-TTGGTCCTTAGCCACTCCTTC-3' and 5’-TGGAGTCCAGCAGACTCAAT-3'; iNOS: 5'-AGCACAGAATGTTCCAGAATCCC-3' and 5’-GTGAAATCCGATGTGGCCTTG-3'; GAPDH: 5'-AGGAGCGAGACCCCACTAACA-3' and 5’-AGGGGGGCTAAGCAGTTGGT-3'.

#### ROS measurement

ROS production in BMDMs was evaluated using ROS sensitive dye 2’,7'-dichlorodihydrofluorescein diacetate (DCFH-DA) [33, 34]. Cells were seeded in a 24 well plate (5 × 10^4^ cells/well) and cultured in DMEM supplemented with 10% FBS and 1% of P/S for 12 h at 37 °C and 5% CO_2_. Then cells were stimulated with 500 ng/mL of LPS for 12 h to obtain M1 phenotype. After stimulation, M1 macrophages were incubated with free NAC solution (122.5 µg/mL), PSLipos (1mM of the total lipid concentration) and PSLipos-NAC dispersion (1mM of the total lipid concentration, containing 122.5 µg/mL NAC) respectively for further 24 h. Next, cells were washed twice with PBS and incubated with 10 µM DCFH-DA in Hanks’ balanced salt solution (HBSS) for 30 min. Relative intracellular fluorescence intensity was measured by a microplate reader (Thermo Fisher, USA) with 485 nm excitation and 530 nm emission after washing the cells with HBSS. The high DCF fluorescence signal implies high intracellular ROS.

#### Anti-inflammatory and pro-healing assays using a transwell coculture system

A co-culture transwell system was established to study the macrophage-mediated anti-inflammatory and pro-healing effects of PSLipos-NAC with different modulus. Briefly, the BMDMs (2 × 10^4^ cells/well) were seeded in the upper chamber and MLE-12 cells (5 × 10^4^ cells/well) were seeded in the lower chamber and cultured for 24 h to reach approximately 80% confluence.

##### Enzyme-linked immunosorbent assay (ELISA)

The BMDMs in the upper chamber and MLE-12 cells in the lower chamber were stimulated with 500 ng/mL LPS for 12 h, respectively, and free NAC solution (122.5 µg/mL), PSLipos (1 mM of the total lipid concentration) and PSLipos-NAC dispersion (1 mM of the total lipid concentration, containing 122.5 µg/mL NAC) respectively were added to the upper chamber of the transwell for another 24 h incubation. The untreated group without LPS and samples was considered as the control. After that, the supernatants of MLE-12 cells in the lower chamber were collected and the inflammatory cytokines including TNF-α, IL-1β and IL-6 were determined by ELISA Kit according to the manufacturer’s protocol (LunChangShuo Biotech, Xiamen, China).

##### NO assay

The nitrite concentration in the culture medium was measured as an indicator of NO production by a modified Griess reagent according to the manufacturer’s instruction [35]. Briefly, the culture supernatant of MLE-12 cells in the lower chamber was collected and 100 µL of these supernatants were mixed with an equal volume of Griess reagent in a 96-well plate and incubated at room temperature for 15 min, and the absorbance was measured at 540 nm using a microplate reader. The amount of nitrite in each sample was calculated from a standard curve prepared using known concentrations of sodium nitrite (NaNO_2_) as the nitrite source.

##### Scratch-wound healing assay

The MLE-12 cell monolayer in the lower chamber was scratched using a 200 µL pipette tip before washing three times with phosphate-buffered saline (PBS) to clear cell debris and floating cells. After that, BMDMs and MLE‑12 cells were cultivated in 2% FBS DMEM and treated with LPS (500 ng/mL) for 12 h, and the BMDMs (in the upper chamber) were then stimulated with free NAC solution (122.5 µg/mL), PSLipos (1mM of the total lipid concentration) and PSLipos-NAC dispersion (1mM of the total lipid concentration, containing 122.5 µg/mL NAC) respectively. At established time points (0 and 24 h) after the samples adding, cells were photographed using a light microscope at the same position of the wound. The scratch-wound area was quantitatively using ImageJ software and migration rate was determined using the formula (2) shown below:

2 $$Migration rate\left(\%\right)=\frac{{{A}_{0}-A}_{t}}{{A}_{0}}\times 100\text{\%}$$

A_0_: The scratch area at 0 h.

A_t_: The scratch area without migrating cells at 24 h.

### In vivo assay for treating ALI

#### Bleomycin‑induced ALI mouse model

Male BALB/C mice (7–8 weeks, 17–22 g, n = 8) were anesthetized and then instilled intratracheally with 50 µL saline alone or bleomycin (2.5 U/kg) diluted in 50 µL sterile saline [36, 37]. After 2 h, the PBS, NAC solution (1.8 mg/mL), PSLipos (15 mM of the total lipid concentration) and PSLipos-NAC (15 mM of the total lipid concentration, containing 1.8 mg/mL NAC) with different modulus were administered into the lungs via aerosol inhalation using a commercial medical air compression nebulizer (Yuwell, 403D) to form inhaled droplets as shown in Fig. S1, which had a median particle size of about 3.9 μm according to the manufacturer’s guidelines. The amount of sample was fixed at 4 mL per mouse. The mice were sacrificed 24 h after the administration, and the lung tissues and serum were harvested for analysis.

#### Histopathological analysis

The lung tissue was fixed in a 4% paraformaldehyde solution and embedded in paraffin, and then cut into 5 μm thick sections. The sections were stained with H&E Staining Kit [38], then monitored by NanoZoomer S360 (Hamamatsu Photonics K.K., Japan). A total of 10 fields at 10× magnification (NDP.view 2 software, Hamamatsu Photonics K.K.) were examined for each histologic slice. The indexes of lung injury were evaluated from the five histological features using a scoring system as Gao et al. described [39, 40]: (1) neutrophils in the alveolar space, (2) neutrophils in the interstitial space, (3) hyaline membranes, (4) proteinaceous debris filling the airspaces, and (5) alveolar septal thickening. Each was scored 0, 1, or 2 according to the injury severity. These five independent variables were weighted based on the relevance to ALI, and then were normalized to the number of fields. The final injury score was a continuous value between 0 and 1.

#### Immunohistochemistry (IHC)

Tissue sections were treated with Anti-iNOS antibody (Abcam) overnight at 4 °C, then washed three times and incubated with a biotinylated secondary antibody in a blocking solution for 1 h. Subsequently, antigen-antibody reactions were detected by staining with diaminobenzidine (Beyotime, China). All sections were imaged using NanoZoomer S360 (Hamamatsu Photonics K.K., Japan). The proportion of iNOS positive area of each field was quantified using Image J software.

#### Real-time quantitative PCR

Total RNA was isolated and purified from the lung tissue using HiPure Total RNA Mini Kit according to the manufacturer’s instruction. Relative gene expression was calculated as described in 2.5.2. The primer sequences used in the experiments were as follows:

TNF-α: 5'-GGCAGGTCTACTTTGGAGTCATTGC-3' and 5’-ACATTCGAGGCTCCAGTGAATTCGG-3'; IL-1β: 5' -TGCCACCTTTTGACAGTGATG-3' and 5’-TGATACTGCCTGCCTGAAGC-3'; IL-6: 5' -TTGGTCCTTAGCCACTCCTTC-3‘and 5’-TGGAGTCCAGCAGACTCAAT-3'; iNOS: 5'-AGCACAGAATGTTCCAGAATCCC-3' and 5’-GTGAAATCCGATGTGGCCTTG-3'; GAPDH: 5'-AGGAGCGAGACCCCACTAACA-3' and 5’-AGGGGGGCTAAGCAGTTGGT-3'.

#### Cytokine analysis

The cytokine levels of the serums were quantified using the ELISA kit according to manufacturer’s instructions. The measured cytokines were TNF-α, IL-1β and IL-6.

### Statistical analysis

All the data were presented as means ± standard error of mean (SEM) from at least three independent experiments or biological replicates (N ≥ 3). Statistical analysis was performed using GraphPad Prism 8 by unpaired t-tests or one-way ANOVA with Bonferroni post hoc test when applicable. P value less than 0.05 was considered to be statistically significant.

## Results and discussion

### Preparation and characterization of PSLipos-NAC

Apoptotic-cell-inspired PSLipos-NAC nanosystems with different modulus were obtained by loading NAC into the corresponding PSLipos using a pH gradient method. As shown in Table 1, the encapsulation efficiency (EE%) and drug loading efficiency (DLE%) of PSLipos-L-NAC were 70.2 ± 0.9% and 19.5 ± 0.2%, respectively, and the EE% and DLE% of PSLipos-H-NAC were 69.9 ± 0.7% and 19.4 ± 0.2%, which were significantly higher than those obtained by thin film hydration and reverse phase evaporation (Fig. S2), and similar to the EE% and DLE% values of 60.1 ± 1.8% and 14.3 ± 0.4% respectively for the dinomenine hydrochloride-loaded liposomes prepared by the pH gradient method, as reported by Shen et al. [41]. The results strongly indicated that the hydrophilic NAC was easily loaded into internal aqueous phase using a transmembrane pH gradient method under mild conditions, and that liposomal elasticity has minimal influence on NAC loading. It was widely accepted that the pH gradient method was less disruptive to drug loading due to lower temperature and no sonication and filtration [42, 43]. Thus, PSLipos-NAC obtained by the pH gradient method was used for subsequent experiments.

**Table 1:** The size, zeta potential, EE% and DLE% of PSLipos and PSLipos-NAC with different modulus

Sample	pH value	Hydrodynamic diameter (nm)	PDI	Zeta potential (mV)	EE%	DLE%
PSLipos-H	7.4 ± 0.1	115.6 ± 1.1	0.207 ± 0.017	-58 ± 1	/	/
PSLipos-L	7.3 ± 0.1	128.2 ± 0.6	0.240 ± 0.004	-69 ± 2	/	/
PSLipos-H-NAC	7.3 ± 0.1	112.6 ± 1.0	0.212 ± 0.021	-54 ± 1	69.9 ± 0.7%	19.4 ± 0.2%
PSLipos-L-NAC	7.3 ± 0.1	127.0 ± 0.9	0.232 ± 0.004	-61 ± 1	70.2 ± 0.9%	19.5 ± 0.2%

**Fig. 1:**
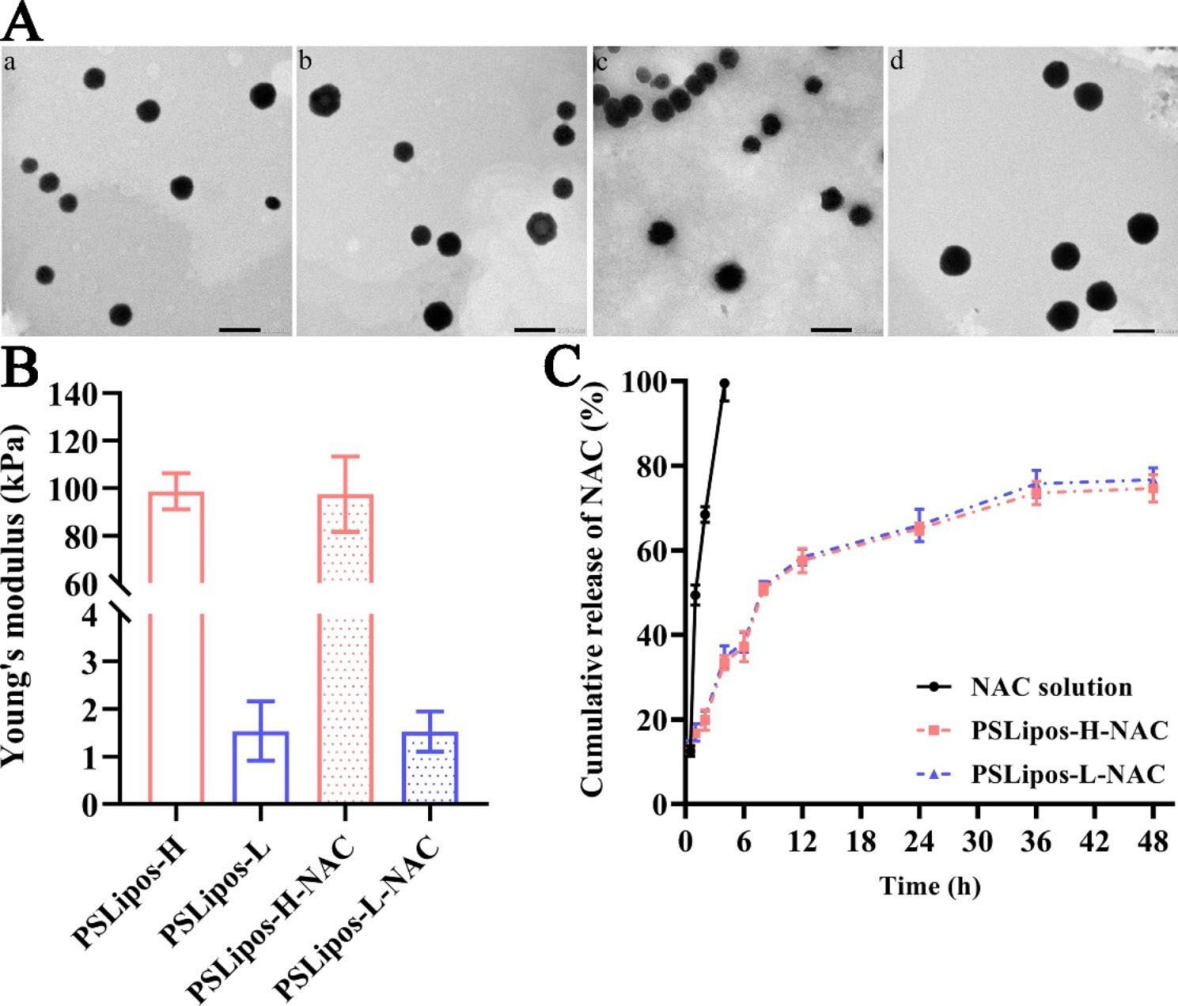
Characterization of PSLipos and PSLipos-NAC. (**A**) Transmission electron microscopy images. a, b, c and d represent PSLipos-H, PSLipos-L, PSLipos-H-NAC and PSLipos-L-NAC, respectively (scale bar = 200 nm). (**B**) Young’s modulus of PSLipos and PSLipos-NAC measured by AFM. (**C**) The in vitro NAC release profiles of free NAC and PSLipos-NAC with different modulus

The physicochemical properties of blank and NAC-loaded PSLipos with different modulus suspensions were then examined in a stable and homogeneous state (Fig. S3). As shown in Table 1, both of them exhibited similar hydrodynamic diameters of approximately 120 nm and PDI of approximately 0.25, as determined via dynamic light scattering, as well as similar negative zeta potential values of approximately − 60 mV, indicating that the hydrophilic NAC encapsulated in the aqueous core of nano-liposomes had little effect on the size and surface potential of liposomal nano-carriers. In the accelerated physical stability experiments, both PSLipos and PSLipos-NAC with different modulus suspensions showed very good stability with almost unchanged pH values, particle sizes, and zeta potentials whether centrifuged at 3200 *g* for 1 h at 25 °C or shaken at 180 beats for 24 h at 37 °C (Tables S2 and S3). And both of them also showed a good long-term physical stability over 30 days under cryogenic conditions (4 °C) (Table S4). The TEM images (Fig. 1A) confirmed that both PSLipos and PSLipos-NAC with different modulus were spherical in morphology with a uniform particle size of approximately 100 nm in a dried state. Nano-liposomal elasticity correlates with the concentration of SDC, which is often utilized as an edge activator to regulate liposome flexibility and deformability [13]. In our work, the blank and NAC-loaded PSLipos containing SPC, DPPS and Chol without SDC (PSLipos-H and PSLipos-H-NAC) showed the higher Young’s modulus of ~ 100 kPa, while the incorporation of about 4.64 wt% SDC into biolayer lipid membranes resulted in more flexible and deformable liposomal nanosystems (PSLipos-L and PSLipos-L-NAC), with the Young’s modulus being lowered to ~ 2 kPa (Fig. 1B), which also demonstrated that nano-liposomal elasticity was less affected by loading aqueous medicines in liposomes. Furthermore, PSLipos-H-NAC and PSLipos-L-NAC exhibited a similar slow-release profile of NAC in the Gamble’s solution (pH 7.4) at 37 °C mimicking lung fluid medium, and almost 30% NAC remained in the nano-liposomes even after 48 h incubation (Fig. 1C), demonstrating that the nano-liposomal elasticity had minimal influence on in vitro NAC release.

### Elasticity influenced the fate of PSLipos-NAC captured by macrophages

**Fig. 2:**
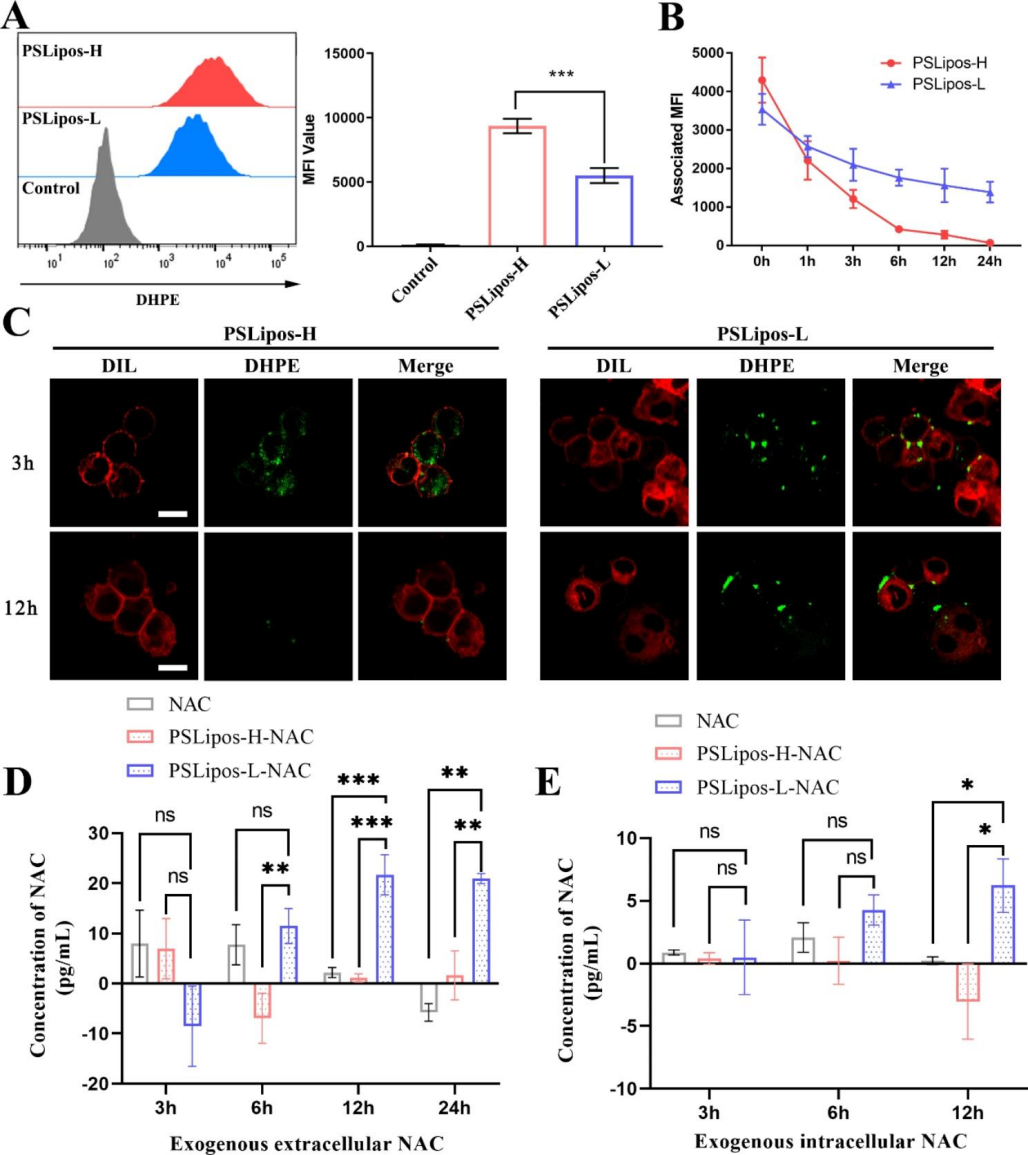
The fate of PSLipos-NAC nanosystems incubated with Raw264.7 cells. (**A**) Flow cytometry analysis of Raw264.7 cells after 24 h incubation with PSLipos-H and PSLipos-L. The MFI value represents the amount of PSLipos captured by the cells. (**B**-**C**) Distribution of PSLipos in Raw264.7 cells. PSLipos were co-cultured with cells for 3 h before being replaced with fresh medium to maintain the culture for a period of time. The MFI values were detected at the indicated times (**B**). The distribution of PSLipos after 3 and 12 h was analyzed by confocal microscopy (**C**). Cell membranes were marked in red by DIL. The scale bar represents 10 μm. (**D**-**E**) After a 3 h co-incubation of cells with PSLipos-H-NAC or PSLipos-L-NAC, fresh culture medium was replaced and cells were further cultured. Extra- and intra-cellular NAC concentrations were tracked over time using a NAC ELISA kit. Background correction was performed for all the measurements to obtain the exogenous NAC from free NAC or PSLipos-NAC treatment by subtracting the endogenous NAC in M1 macrophages at the corresponding time point, ns represented no significant difference, *p < 0.05, **p < 0.01, ***p < 0.001. The results represented the mean values ± SD. (n>3)

The macrophage-mediated therapeutic effects of drug-loaded nanotherapeutic systems are significantly dependent on their nano-cell surface interactions and intracellular fate in macrophages, which would affect the mode of drug action at the cellular level. In this work, the fluorescence-labeling method was used for monitoring the effect of PSLipos elasticity on the cellular capture and intracellular fate of PSLipos-based nanosystems in macrophages. Fluorescence measurements via flow cytometry demonstrated an about 40% reduction in PSLipos-L capture by RAW264.7 cells as compared to PSLipos-H (Fig. 2A), indicating that lower modulus impeded PSLipos-L nanosystems capture by macrophages, which was in good agreement with previous report by Key et al. on the resistance of soft nanoconstructs to macrophage capture [44]. Interestingly, from the time course of the fluorescent signal intensity changes on RAW264.7 cells capturing PSLipos (Fig. 2B), it could be seen that the signal intensity for PSLipos-H quickly dropped to almost zero in contrast to the slow decrease of about 50% for PSLipos-L within 24 h, indicating that PSLipos elasticity greatly affected their lifespan in macrophages, and PSLipos-L with lower modulus had a longer lifespan in macrophages. Further observation by confocal microscopy revealed that PSLipos-L was mostly found to be still attached to the outer surface of macrophage membrane up to 12 h incubation, whereas PSLipos-H was found to be internalized into the cytoplasm after 3 h incubation and almost disappeared after 12 h due to the degradation in macrophages (Fig. 2C). Additionally, after treating cells with NAC or NAC-loaded PSLipos with different modulus for 3 h, fresh culture medium was replaced and the extra- and intra-cellular NAC concentrations were tracked over time (< 24 h) using a NAC ELISA kit. To exclude interference from endogenous NAC in experiments as much as possible, which may also depend on the state of macrophage polarization, background correction was performed for all the measurements to obtain the exogenous NAC from free NAC or PSLipos-NAC treatment by subtracting the endogenous NAC in M1 macrophages at the corresponding time point. It was found that exogenous extracellular NAC concentrations in PSLipos-L-NAC group showed no significant differences with free NAC group at 3 and 6 h, and PSLipos-H-NAC group at 3 h, then they were significantly higher than the other two groups till 24 h (Fig. 2D). As for exogenous intracellular NAC concentrations (Fig. 2E), PSLipos-L-NAC group showed no significant differences with free NAC and PSLipos-H-NAC groups at 3 and 6 h, but then remarkably higher than them at 12 h. Obviously, after PSLipos-L-NAC and PSLipos-H-NAC with similar in vitro NAC release properties were captured by macrophages, they would exhibit different local NAC release behavior targeting to macrophages due to their modulus-dependent interactions with macrophages. Considering that NAC had a short half-life in vivo [45], and PSLipos-H showed relatively quick internalization and metabolism by the macrophages while PSLipos-L continuously adhered on the macrophage surface based on their cellular distribution (Fig. 2C), PSLipos-L-NAC may more effectively modulate macrophages than free NAC and PSLipos-H-NAC possibly due to the direct and indirect diffusion of their sustained-releasing NAC into the cells. The detailed mechanism of small or macromolecular drugs from PSLipos-L bound to macrophage surface into macrophages is still not fully understood, which is being investigated by us using the fluorescence technique. These findings suggested that the intracellular fate of PSLipos-based nanotherapeutics captured by macrophages is influenced by their elasticity, and that lower modulus may be advantageous in prolonging their lifetime within macrophages, enabling gradual NAC release and sustained targeting therapeutic effects.

Soft nanoparticles are typically assumed to interact with macrophages transiently and resist macrophages phagocytosis. Palomba et al. reported that low-modulus nanoparticles (~ 100 kPa) interacted only transiently (< 30 s) with Raw264.7 cell membranes through high-resolution live cell imaging, resulting in less endocytosis than higher modulus nanoparticles (10 MPa) [46]. However, the synergistic effects of softness and ligand-receptor interactions of active nanoparticles on macrophages function were frequently overlooked. Our previous research has demonstrated that receptor-mediated soft nanoparticles were resistant to internalization and had a substantial amount of time to bind to macrophage membranes [13]. In this work, our findings further revealed that tailoring the softness of ligand-directed active macrophage-targeting nanoparticle-based drug delivery systems may provide a potential approach to modulate the mode of drug action at a cellular level for better therapeutic performance.

### Elasticity influenced the anti-inflammatory effects of PSLipos-NAC on macrophages

**Fig. 3:**
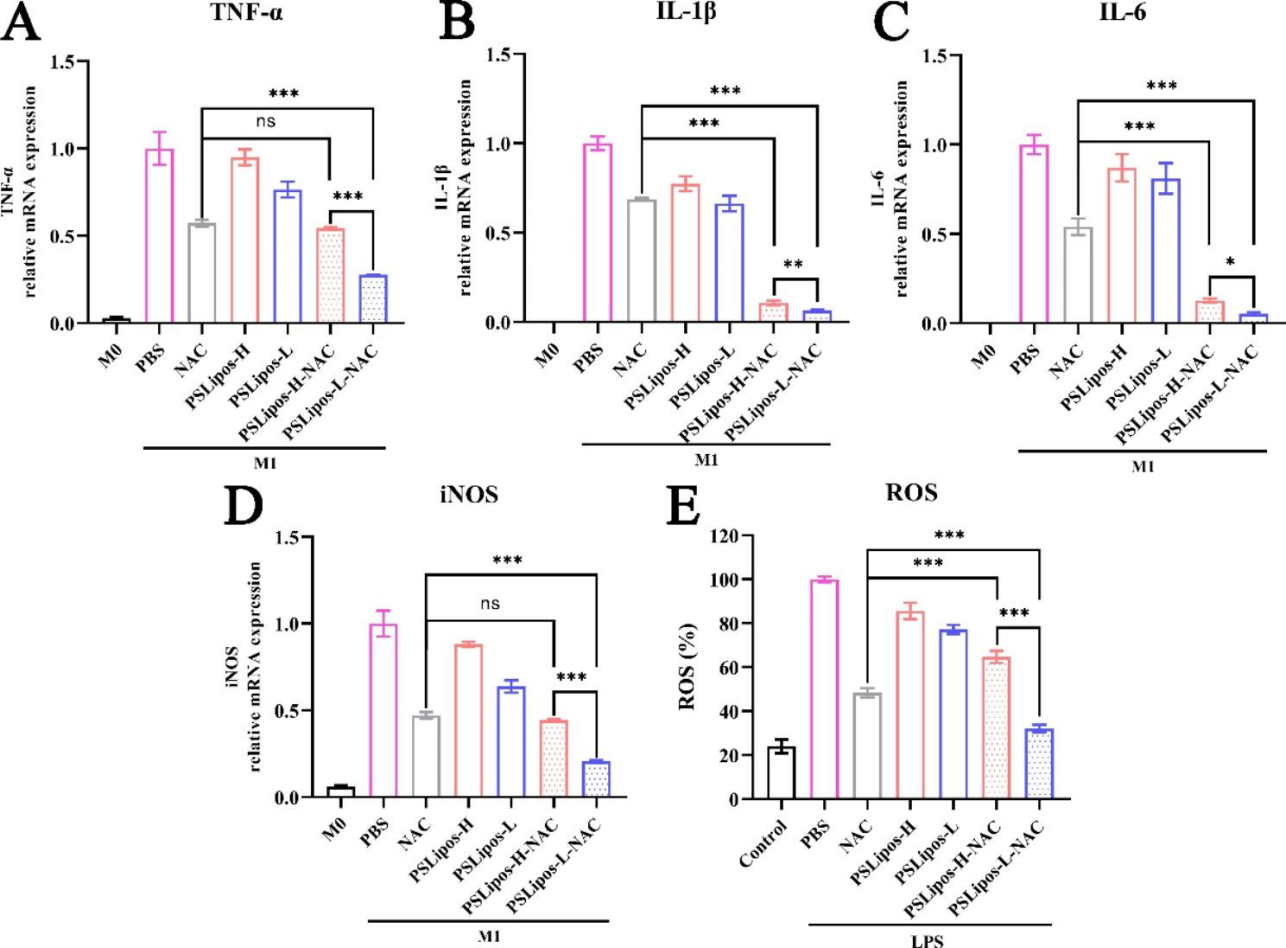
PSLipos-L-NAC had a stronger anti-inflammatory effect on M1 macrophages. The mRNA expression levels of pro-inflammatory cytokines of M1 macrophages treated with NAC, PSLipos and PSLipos-NAC respectively were analyzed by real-time quantitative PCR. (**A**) TNF-α, (**B**) IL-1β, (**C**) IL-6, (**D**) iNOS. (**E**) ROS production in M1 macrophages was evaluated by DCFH-DA. ns represented no significant difference, *p < 0.05, **p < 0.01, ***p < 0.001. The results represented the mean values ± SD. (n = 3)

We further investigated the elasticity of PSLipos-NAC on the inflammatory response of lipopolysaccharide (LPS)-treated BMDMs (M1 macrophages) after confirming their non-cytotoxicity to BMDMs using the CCK-8 assay (Fig. S4). The LPS-induced M1-like BMDMs were treated with NAC, blank PSLipos and PSLipos-NAC with different modulus in the presence of LPS for 24 h, and then the representative inflammatory cytokine mRNA expression levels including TNF-α, IL-1β and IL-6, and iNOS were analyzed (Fig. 3A and D). As expected, LPS (500 ng/mL) induced higher expression levels of these inflammatory mRNAs compared with those in untreated cells, indicating the successful induction of M1 macrophages. NAC treatment could inhibit these inflammatory mRNA expression levels in M1 type BMDMs with the inhibition rate of approximately 30%~50%, which were consistent with previous results reported by Palacio et al. [47, 48]. As blank macrophage-targeting liposomal nano-carriers, PSLipos-H or PSLipos-L alone also suppressed the expression of inflammatory genes of TNF-α, IL-1β, IL-6 and iNOS in a certain extent, suggesting that PSLipos with phosphatidylserine exposed on the liposomal surface could mimic apoptotic cells to inhibit the synthesis of inflammatory factors in LPS-stimulated macrophages [49], and PSLipos-L showed a stronger anti-inflammatory effects than PSLipos-H probably resulting from their long-term binding to the PS receptors on the macrophage surface, though their anti-inflammatory effects seemed to be weaker than those of NAC. As for NAC-containing PSLipos, the decrease of inflammatory gene expression in M1 macrophages was more pronounced, especially for PSLipos-L-NAC with relatively low macrophage capture efficiency, which inhibited around 70%~90% of TNF-α, IL-1β, IL-6, and iNOS mRNA expression in M1 macrophages even more effectively than PSLipos-H-NAC with high macrophage capture efficiency. Meanwhile, PSLipos-H as a highly macrophage-targeting nanocarrier almost did not improve the inhibition effect on TNF-α and iNOS mRNA expression of NAC in M1 macrophages.

We also investigated the effect of PSLipos-NAC on the formation of ROS in M1 macrophages. It is known that the formation of ROS is a normal physiological metabolic process which happens in every cell, which can serve as an important short-lived second messenger in cell signaling mainly by oxidizing the thiol group in cysteine residues of a protein. However, excessive ROS under inflammation can overcome antioxidant defenses leading to oxidative stress, and consequently cause the damage of intracellular constituents such as lipids, proteins, and DNA. Furthermore, high levels of ROS can activate pro-inflammatory transcription factors such as NF-κB that up-regulate expression of pro-inflammatory chemokines/cytokines related to M1 macrophage phenotype [50, 51]. We found that the increased ROS level in M1 type BMDMs was significantly reduced after treatment with NAC, blank PSLipos and PSLipos-NAC with different modulus respectively. More interestingly, the anti-oxidative effect of PSLipos-L-NAC on M1 macrophages was more effective than that of free NAC, which could decrease the ROS level in LPS-induced M1 macrophage close to the control M0 macrophage, while the anti-oxidative effect of highly macrophage-targeting PSLipos-H-NAC was lower than that of free NAC (Fig. 3E), possibly due to the quick degradation of PSLipos-H-NAC in macrophages.

The above results of macrophage responses induced by PSLipos-NAC with different modulus strongly suggested that the elasticity of ligand-directed macrophage-targeting nanoparticle-based drug delivery systems significantly influence their regulating on macrophage activities, and a higher capture efficiency does not necessarily mean a better effect, while modulus-dependent intracellular fate of nanotherapeutics may greatly affect the effects of loaded drugs on macrophages at the cellular level. Low-modulus PSLipos-L-NAC can target macrophages and keep durable binding to macrophage surfaces, exerting an enhanced PS-receptor-mediated anti-inflammatory activity meanwhile allowing a slow release of loaded NAC for synergistic inhibition of macrophage inflammatory responses.

### PSLipos-L-NAC effectively suppressed macrophage inflammatory responses to promote lung epithelial wound healing

**Fig. 4:**
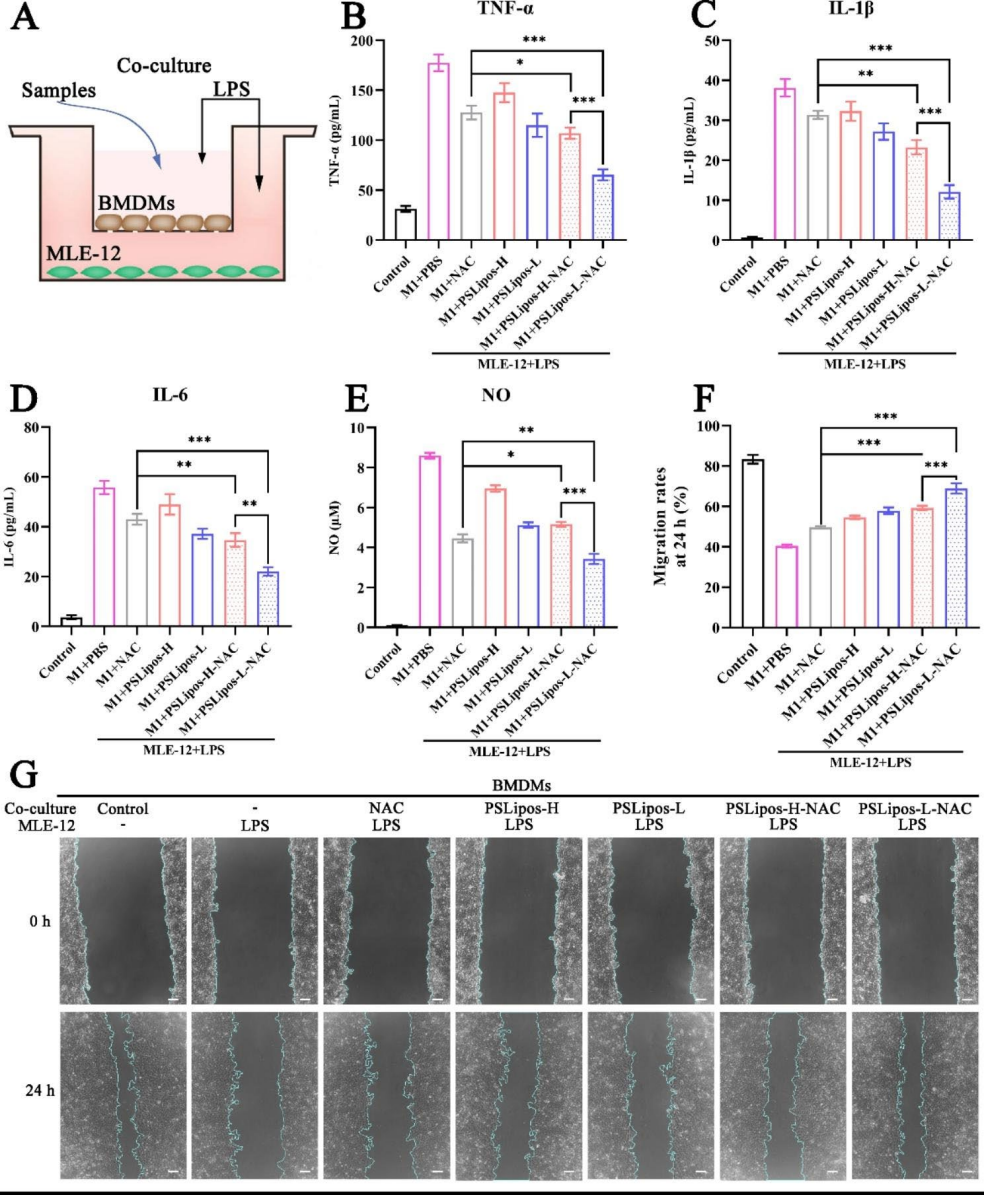
PSLipos-L-NAC more effectively improved wound healing in lung epithelial cells via macrophage-mediated way. (**A**) Schematics of inflammatory BMDMs and MLE-12 co-cultured system. The pro-inflammatory cytokines expression of (**B**) TNF-α, (**C**) IL-1β, (**D**) IL-6 in co-culture system was detected after treating with samples using ELISA (n>3). (**E**) NO production. (**F**) and (**G**) Scratch wound assay was used to assess the wound healing of MLE-12 cells under inflammatory environment. Scale bars, 100 μm. Data are from three independent experiments and presented as the mean ± SD. (ns represented no significant difference, *p < 0.05, **p < 0.01, ***p < 0.001)

Macrophage dysfunction that causes severe inflammatory response and even tissue injury is critical in the initiation and progression of inflammation in ALI, and hence macrophage has been recognized as a potential therapeutic target to maintain immune homeostasis in the lung to treat ALI [19]. Herein, we established an in vitro co-culture model of BMDMs and lung epithelial MLE-12 cells to investigate the role of PSLipos-NAC in promoting macrophage anti-inflammatory and pro-healing responses (Fig. 4A). The co-culture system of BMDM and MLE-12 cells were stimulated with LPS for 12 h to mimic the active state of the cells in an inflammatory environment in pulmonary lesions, then NAC, blank PSLipos and PSLipos-NAC with different modulus were added to the upper chamber containing M1 type BMDMs of the transwell, respectively. The culture supernatant of MLE-12 cells in the lower chamber were collected to detect the expression of inflammatory factors and nitric oxide (NO) production. As shown in Fig. 4, LPS enhanced TNF-α, IL-1β, and IL-6 protein levels expression, which were then inhibited by NAC, blank PSLipos and PSLipos-NAC at different extent. Among them, PSLipos-L-NAC showed the highest inhibition rates, which were ~ 63% for TNF-α, ~ 68% for IL-1β, and ~ 60% for IL-6 (Fig. 4B and D). NO contributes importantly to the pathophysiology of ALI [52]. LPS stimulation led to increase NO generation in MLE-12 cells, compared to control cells (Fig. 4E). In comparison to the PBS-treated M1 macrophage group, NO generation was reduced by ~ 50%, ~ 40%, and ~ 20% in cells treated with free NAC, blank PSLipos-L, and PSLipos-H, respectively. And the reduction in NO levels following PSLipos-L-NAC treatment was around 60%, which was much greater than the 40% reduction in NO levels after PSLipos-H-NAC treatment. The results evidently revealed that the softness of PSLipos-NAC was beneficial to their macrophage-mediated regulation on the inflammatory micro-environment of MLE-12 cells.

Furthermore, the scratch wound healing assay was used to determine the macrophage-mediated pro-healing effects of PSLipos-NAC on lung epithelial tissue by monitoring the scratch-wound healing rate of MLE-12 cell monolayer in the lower chamber with BMDMs in the upper chamber of the transwell. As shown in Fig. 4F and G, the migration rate of MLE-12 cell was 83 ± 2% for the control group without LPS treatment after scratching wound for 24 h, but it was significantly decreased to approximately 50% of the control group in the LPS-treated MLE-12 group. It was confirmed that the inflammatory micro-environment would lead to impaired proliferation and migration of MLE-12 cells. Although inflammation itself is inherently a protective response, persistent inflammatory response would lead to epithelial cells dysfunction, including abnormal proliferation and differentiation, which delays the lung wound-healing process [53]. The healing of wound gap in the inflammatory MLE-12 cell monolayer was increased by treating BMDMs with NAC, PSLipos alone and PSLipos-NAC with different modulus, respectively, and the PSLipos-L-NAC treatment was responsible for the highest migration rate of nearly 70%. These results strongly indicated that PSLipos-L-NAC with low modulus could significantly promote the proliferation and migration of lung epithelial cells in inflammatory micro-environment possibly via synergistically inhibiting the inflammatory responses of macrophages.

### PSLipos-L-NAC inhalation remarkably reduced inflammatory responses and improved lung tissue repair in the ALI mouse

**Fig. 5:**
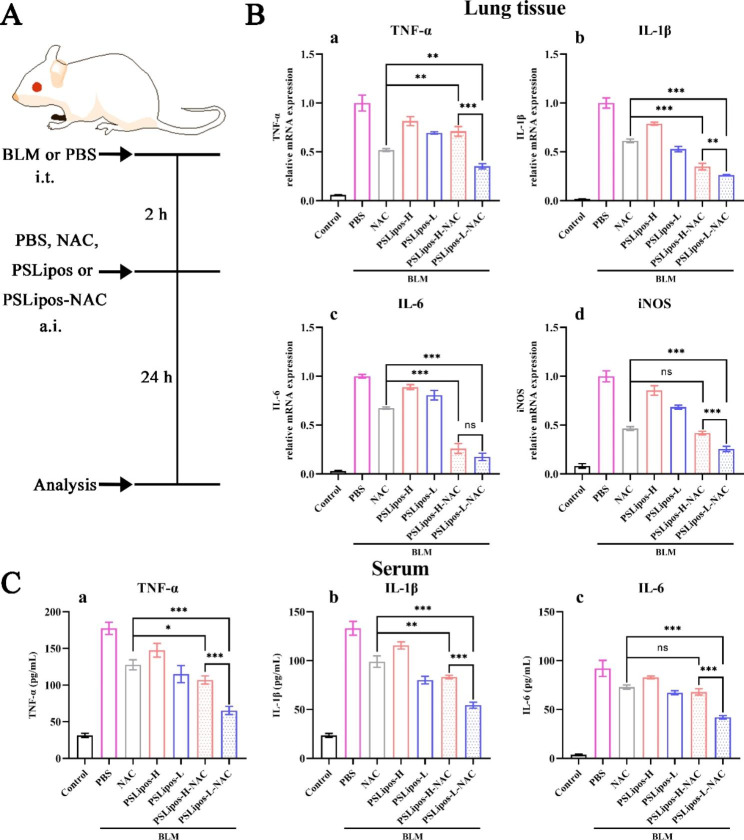
Anti-inflammatory effects of inhaled PSLipos-NAC nanosystems in a bleomycin-induced ALI mouse model. (**A**) Timeline of experiments. The lung tissue and serums were harvested to assess the expression of pro-inflammatory factors at 24 h after treatment. i.t. represented intratracheal injection and a.i. represented aerosol inhalation. (**B**) Expression of pro-inflammatory genes in lung tissue. (**C**) Expression of inflammatory factors in serum. (n ≥ 6 per group, ns represented no significant difference, *p < 0.05, **p < 0.01, ***p < 0.001)

We further adopted a bleomycin-induced ALI mouse model mimicking the acute inflammatory phase of ALI to investigate the therapeutic effects of inhaled PSLipos-NAC on ALI in vivo compared with NAC and PSLipos (Fig. 5A). It should be noted that inhalation of NAC has been world-widely used in the management of ALI/ARDS. The pulmonary inflammation was assessed by analyzing the pro-inflammatory genes and cytokine secretion in the lung tissue and serum respectively. The over-expressed pro-inflammatory genes of TNF-α, IL-1β, IL-6 and iNOS in lung tissue of BLM-induced ALI mouse were significantly down-regulated after the inhalation of NAC, PSLipos and PSLipos-NAC respectively (Fig. 5B). In particular, PSLipos-NAC exhibited a modulus-dependent inhibition on this inflammatory gene expression, and the PSLipos-L-NAC group was found to have the lowest expression levels of approximately 20% ~ 40% of those in BLM-induced group (Fig. 5B). In terms of inflammatory cytokine levels in mice serum, pulmonary administration of PSLipos-L-NAC also provided the best anti-inflammatory effect on the expression of TNF-α, IL-1β and IL-6. Both of the inhibition of inflammatory factors in the lung tissue and serum by PSLipos-NAC with different modulus were well consistent with the in vitro results.

**Fig. 6:**
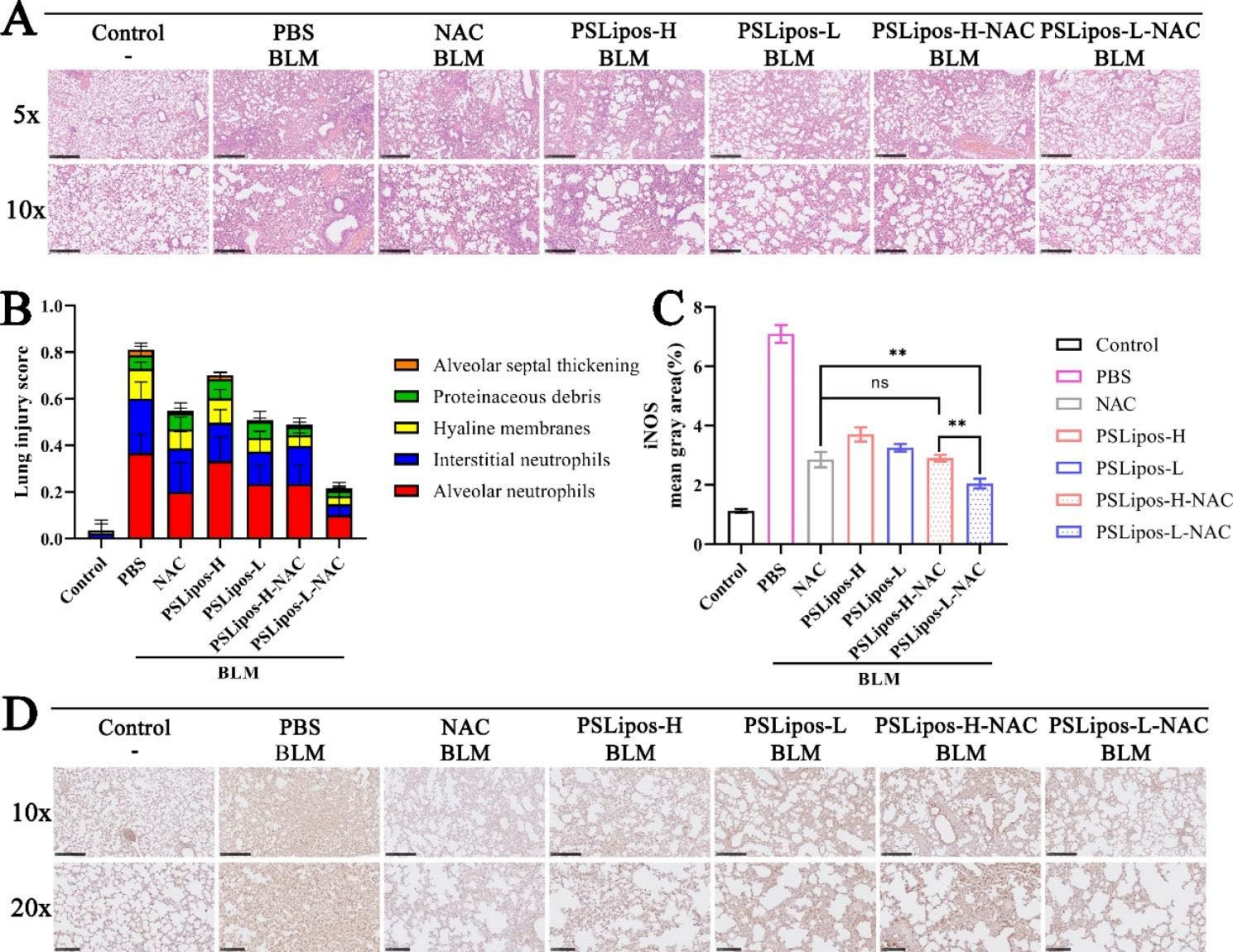
In vivo therapeutic effects of inhaled PSLipos-NAC nanosystems on ALI. (**A**) The representative histological images of H&E-stained lung sections. The scale bar in the upper panel = 500 μm while that in the lower panel = 250 μm. (**B**) The lung injuries were accessed by 5 pathophysiological features to obtain the total injury score. (**C**-**D**) Immunohistochemistry to detect the expression of iNOS in lung tissues.The scale bar in the upper panel = 250 μm while that in the lower panel = 100 μm (**C**). And iNOS was quantified by ImageJ (**D**). (n ≥ 6 per group, ns represented no significant difference, *p < 0.05, **p < 0.01, ***p < 0.001)

The anti-inflammatory and pro-healing efficacy of PSLipos-NAC inhalation in ALI model was also evaluated using pathological tissue sections. H&E staining of pulmonary sections in each experimental group showed an obvious histopathological difference (Fig. 6A). The control group exhibited uniform and small alveoli throughout the visual field, but in the BLM-treated group, the alveolar spaces were significantly occluded, the thickness of the trachea increased, and the pulmonary fibers increased slightly. After the inhalation of PSLipos-L-NAC, gradual clear alveolar walls and attenuate trachea thickness were evidently observed (Fig. 6A), while NAC, PSLipos alone, or PSLipos-H-NAC inhalation exhibited less therapeutic benefits in comparison to PSLipos-L-NAC. The pathological hallmark of ALI is diffuse alveolar damage, characterized by neutrophils accumulation in the alveolar space and interstitial space, hyaline membrane formation, proteinaceous debris filling the airspaces and alveolar septal thickening [40], and the lung injury score can be obtained based on these features. It was found that PSLipos-L-NAC inhalation therapy, in particular, resulted in fewer interstitial and alveolar neutrophils, reduced widespread alveolar damage and thickness of alveolar walls, more effectively than either NAC, PSLipos alone, or PSLipos-H-NAC treatment, displaying the lowest average injury score (Fig. 6B).

The iNOS is a hallmark molecule of M1 macrophages and is generally considered as an activator in accelerating the inflammation process [54]. Immunohistochemical staining (Fig. 6C and D) showed that an increased expression of iNOS were observed in BLM-induced groups compared to the control group, indicating the polarization of M1 macrophages induced by BLM, while PSLipos-L-NAC inhalation showed the most effective inhibitory effect on the expression of iNOS in all the treatment groups due to their synergistically suppressing pro-inflammatory M1 macrophage polarization in lung macrophages. It was obvious that pulmonary admistration of PSLipos-NAC exhibited a modulus-dependent therapeutic effects on a bleomycin-induced ALI mouse model, low-modulus PSLipos-L-NAC could more effectively attenuate the BLM-induced ALI by synergistically inhibiting pro-inflammatory M1 macrophage polarization and inflammatory response than the PSLipos-H-NAC.

## Conclusion

Inhalable *N*-acetylcysteine encapsulated apoptotic-cell-inspired PS-containing nano-liposomes with different modulus were successfully prepared by a method of combinating of the thin-film hydration, ultrasonication and extrusion with pH gradient method for loading *N*-acetylcysteine. The obtained macrophage-targeting PSLipos-NAC displayed modulus-dependent cellular capture, intracellular fate and local NAC release behavior in macrophages, as well as macrophage-mediated anti-inflammatory and pro-healing effects. Low-modulus PSLipos-L-NAC with low capture efficiency of macrophages but durable binding to PS-receptor on the macrophage surfaces induced more effective anti-inflammatory and pro-healing effects, probably due to their long-term effects of PS-receptor-mediation and gradual release of NAC, even better than high-modulus one with higher macrophage capture efficiency but rapid elimination rate. Using in vitro co-culture inflammatory model of BMDMs and MLE-12 cells as well as the BLM-induced ALI mouse model, PSLipos-L-NAC had been proven to effectively suppress macrophage inflammatory responses and attenuate lung injury, providing a potential nano-inhalation therapy for ALI. These findings also evidently confirmed that elasticity of macrophage-targeting nanotherapeutics plays an important role in regulating their therapeutic performance.

## Electronic supplementary material

Below is the link to the electronic supplementary material.

Supplementary Material 1

## Data Availability

Data will be made available on request.

## Abbreviations

ALI: Acute lung injury
ARDS: Acute respiratory distress syndrome
AFM: Atomic force microscope
BLM: Bleomycin
BMDMs: Bone marrow derived macrophages
Covid-19: Coronavirus Disease 2019
Chol: Cholesterol
DCFH-DA: 2',7'-dichlorodihydrofluorescein diacetate
DLE: Drug loading efficiency
DPPS: 1, 2-dipalmitoyl-sn-glycero-3-phospho-L-serine
DMEM: Dulbecco’s modified Eagle medium
EE: Encapsulation efficiency
ELISA: Enzyme-linked immunosorbent assay
FBS: Fetal bovine serum
GAPDH: Glyceraldehyde 3-phosphate dehydrogenase
HBSS: Hanks’ balanced salt solution
IL-1: Interleukin 1
LPS: Lipopolysaccharides
MFI: Mean fluorescence intensity
MLE-12: Mouse lung epithelial 12
NAC: *N*-acetylcysteine
NO: Nitric oxide
PS: Phosphatidylserine
PSLipos: Phosphatidylserine - containing nano-liposomes
PSLipos-L and -H: PS-containing nano-liposomes with low and high modulus
PDI: Polydispersity index
P/S: Penicillin-streptomycin
ROS: Reactive oxygen species
SARS-CoV-2: Severe acute respiratory syndrome coronavirus-2
SDC: Sodium deoxycholate
SPC: Soybean phosphatidylcholine
TEM: Transmission electron microscopy
TNF-α: Tumor necrosis factor α
