# Supplementary material for: Softness enhanced macrophage-mediated therapy of inhaled apoptotic-cell-inspired nanosystems for acute lung injury
Source: J Nanobiotechnology. 2023 May 30;21:172. doi: 10.1186/s12951-023-01930-2 (PMC10226883; doi:10.1186/s12951-023-01930-2)
Supplement: Supplementary file 1 — Supplementary Material 1 [file 12951_2023_1930_MOESM1_ESM.docx]

Supplementary information for:

**Softness enhanced macrophage-mediated therapy of inhaled apoptotic-cell-inspired nanosystems for acute lung injury**

*Dazheng Sun^1#^, Guanglin Zhang^1,3#^, Mingyang Xie^1^, Yina Wang^2^, Xiangchao Liang^1^, Mei Tu^1^, Zhijian Su^2^*, Rong Zeng^1^**

*^1^ Department of Materials Science and Engineering, College of Chemistry and Materials, Jinan University, Guangzhou, 510632,* *P. R. China*

*^2^ Guangdong Provincial Key Laboratory of Bioengineering Medicine, Department of Cell Biology, Jinan University, Guangzhou, 510632, P. R. China*

*^3^ Henry Fok Colloge of Biology and Agriculture, Shaoguan University, Shaoguan, 512005, P. R. China*

* Corresponding authors.

E-mail address: tzengronga@jnu.edu.cn (Rong Zeng); tjnuszj@jnu.edu.cn (Zhijian Su)

# Represents Dazheng Sun and Guanglin Zhang contributed equally to this work.

Table of Contents

[Fig. S1. Typical photo of aerosol inhalation treatment for ALI mice using a micro-nebulizer. 3](#_Toc126427626)

[Fig. S2. Encapsulation efficiency (EE%) and drug loading efficiency (DLE%) of PSLipos-NAC prepared by three different methods (n=5). 4](#_Toc126427627)

[Fig. S3. Typical photo of liposomal suspensions, a, b, c and d represent PSLipos-H, PSLipos-L, PSLipos-H-NAC and PSLipos-L-NAC, respectively. 4](#_Toc126427628)

[Fig. S4. Viabilities of BMDMs and MLE-12 cells after treated with various concentrations of apoptotic-cell-inspired nano-liposomes for 48 h. 5](#_Toc126427629)

[Table S1. The chemical composition of Gamble's solution. 6](#_Toc126427630)

[Table S2. Standard accelerated testing of liposomes (3200 *g*, 25 ℃, 1 h). 7](#_Toc126427631)

[Table S3. Standard accelerated testing of liposomes (180 beats, 24 h, 37 ℃). 8](#_Toc126427632)

[Table S4. Standard long-term testing of liposomes (4 ℃). 9](#_Toc126427633)

# Fig. S1. Typical photo of aerosol inhalation treatment for ALI mice using a micro-nebulizer.

**
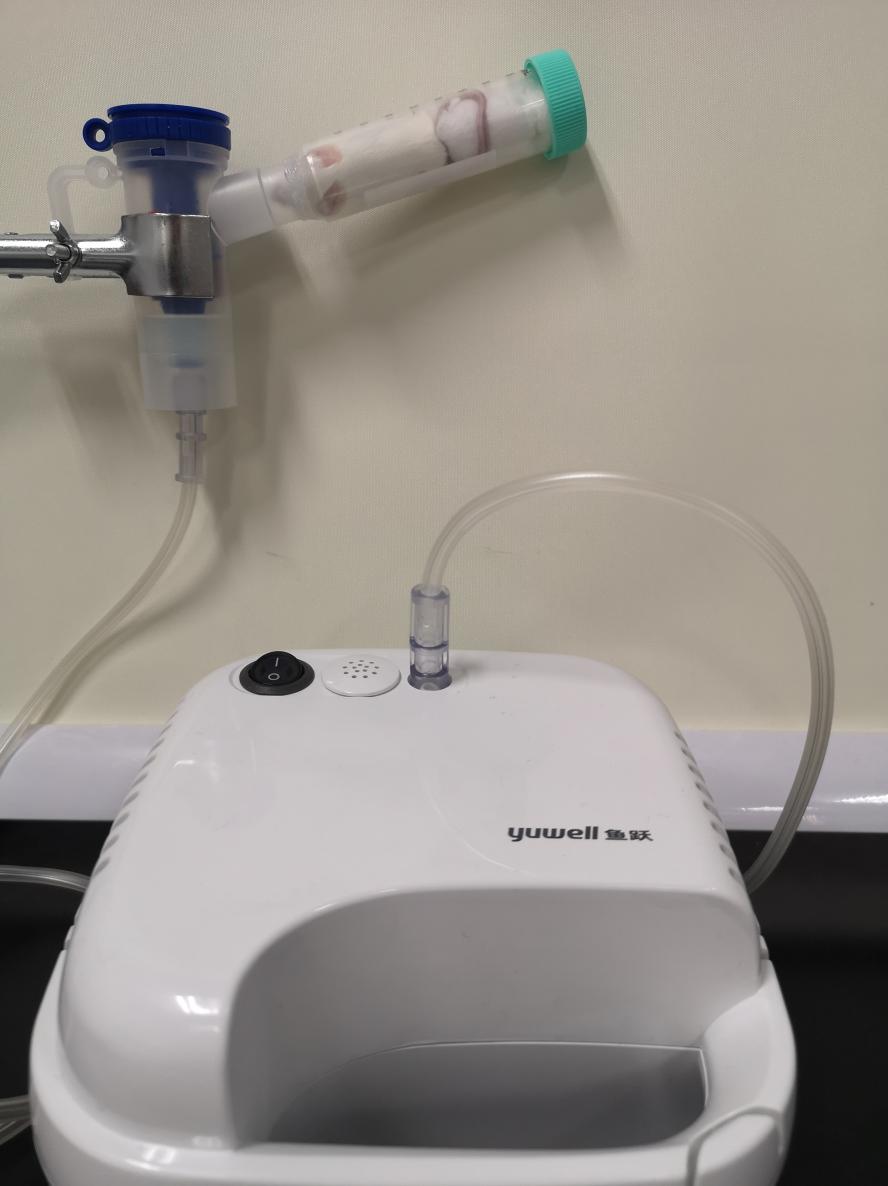
**

# Fig. S2. Encapsulation efficiency (EE%) and drug loading efficiency (DLE%) of PSLipos-NAC prepared by three different methods (n=5).


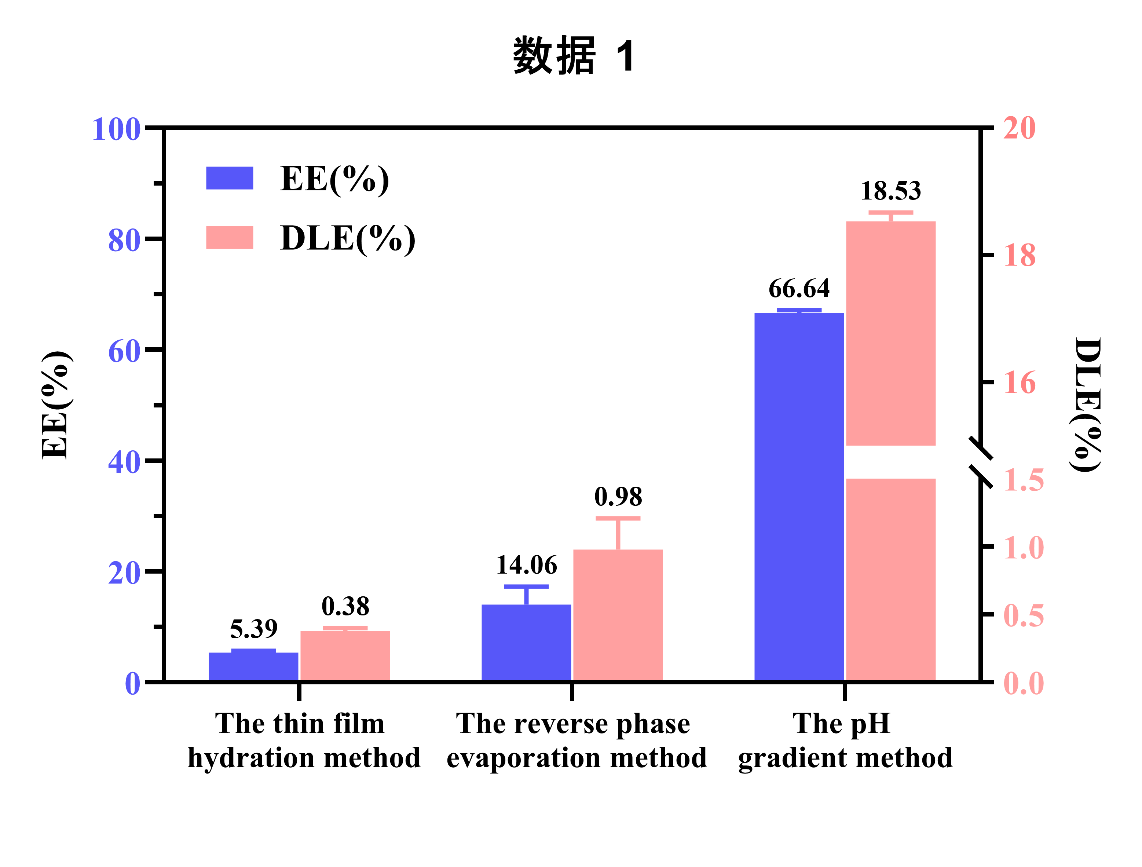


# Fig. S3. Typical photo of liposomal suspensions, a, b, c and d represent PSLipos-H, PSLipos-L, PSLipos-H-NAC and PSLipos-L-NAC, respectively.


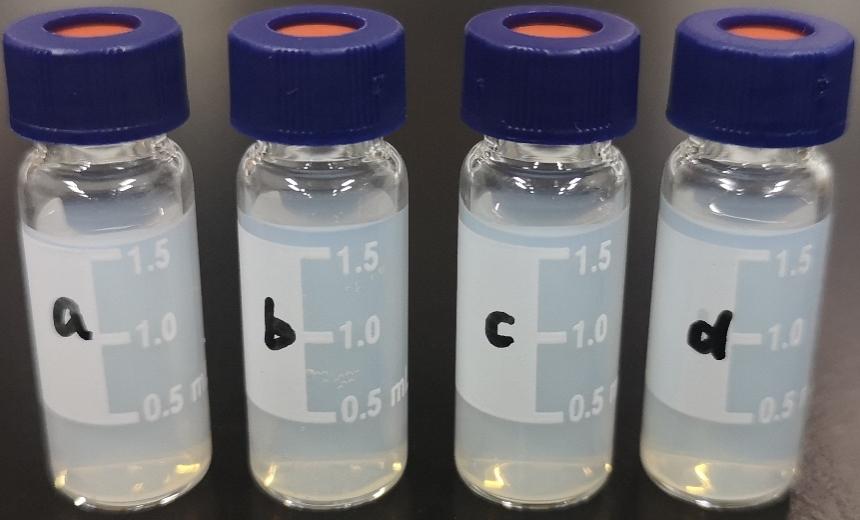


# Fig. S4. Viabilities of BMDMs and MLE-12 cells after treated with various concentrations of apoptotic-cell-inspired nano-liposomes for 48 h.


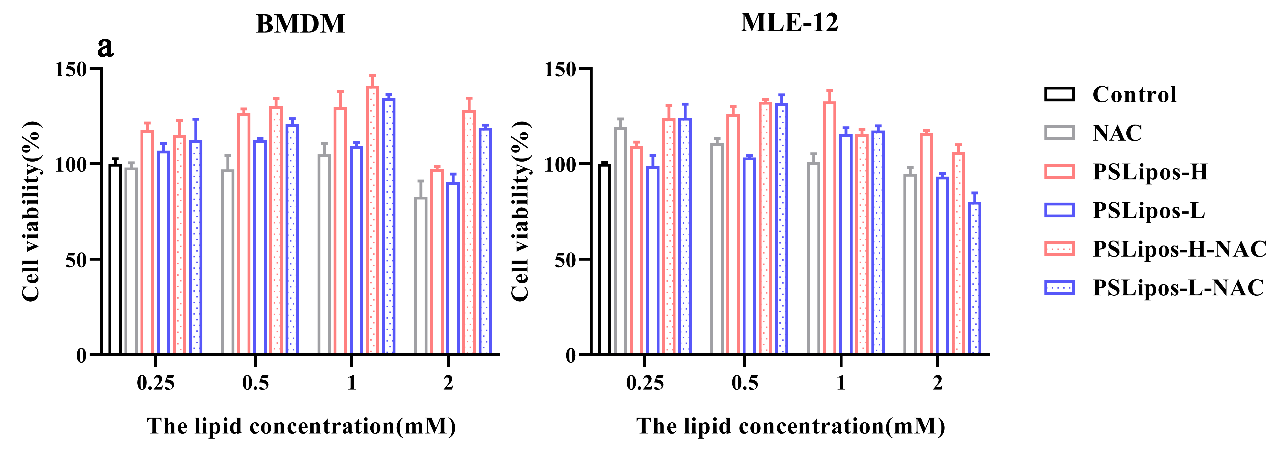


# Table S1. The chemical composition of Gamble's solution.

| Chemicals | Gamble's solution (pH=7.4, g/L) |
| --- | --- |
| MgCl_2_ | 0.095 |
| NaCl | 6.019 |
| KCl | 0.298 |
| Na_2_HPO_4_ | 0.126 |
| Na_2_SO_4_ | 0.063 |
| CaCl_2_·2H_2_O | 0.368 |
| C_2_H_3_O_2_Na | 0.574 |
| NaHCO_3_ | 2.604 |
| C_6_H_5_Na_3_O_7_·2H_2_O | 0.097 |

# Table S2. Standard accelerated testing of liposomes (3200 *g*, 25 ℃, 1 h).

| Sample | | PSLipos-H | PSLipos-L | PSLipos-H-  NAC | PSLipos-L-  NAC |
| --- | --- | --- | --- | --- | --- |
| Before | pH value | 7.4±0.3 | 7.4±0.1 | 7.3±0.2 | 7.3±0.1 |
|  | Hydrodynamic diameter (nm) | 115.6±1.1 | 128.2±0.6 | 112.6±1.0 | 127.0±0.9 |
|  | PDI | 0.207±0.017 | 0.240±0.004 | 0.212±0.021 | 0.232±0.004 |
|  | Zeta potential  (mV) | -58.4±0.8 | -69.1±1.6 | -53.5±1.3 | -61.3±1.3 |
| After | pH value | 7.3±0.4 | 7.4±0.1 | 7.3±0.2 | 7.3±0.1 |
|  | Hydrodynamic diameter (nm) | 112.5±0.9 | 121.2±1.5 | 110.9±2.0 | 126.1±1.5 |
|  | PDI | 0.206±0.012 | 0.228±0.012 | 0.204±0.023 | 0.235±0.006 |
|  | Zeta potential  (mV) | -51.2±1.3 | -65.8±1.1 | -48.2±3.6 | -59.6±3.8 |

# Table S3. Standard accelerated testing of liposomes (180 beats, 24 h, 37 ℃).

| Sample | | PSLipos-H | PSLipos-L | PSLipos-H-  NAC | PSLipos-L-  NAC |
| --- | --- | --- | --- | --- | --- |
| Before | pH value | 7.4±0.3 | 7.4±0.1 | 7.3±0.2 | 7.3±0.1 |
|  | Hydrodynamic diameter (nm) | 115.6±1.1 | 128.2±0.6 | 112.6±1.0 | 127.0±0.9 |
|  | PDI | 0.207±0.017 | 0.240±0.004 | 0.212±0.021 | 0.232±0.004 |
|  | Zeta potential  (mV) | -58.4±0.8 | -69.1±1.6 | -53.5±1.3 | -61.3±1.3 |
| After | pH value | 7.3±0.4 | 7.4±0.2 | 7.3±0.5 | 7.4±0.1 |
|  | Hydrodynamic diameter (nm) | 115.9±1.7 | 130.9±1.4 | 108.8±1.0 | 127.5±3.5 |
|  | PDI | 0.212±0.022 | 0.227±0.012 | 0.201±0.035 | 0.217±0.027 |
|  | Zeta potential  (mV) | -49.6±4.0 | -46.5±7.5 | -38.5±4.4 | -49.5±3.2 |

# Table S4. Standard long-term testing of liposomes (4 ℃).

| Sample | Time (d) | 0 | 5 | 10 | 20 | 30 |
| --- | --- | --- | --- | --- | --- | --- |
| PSLipos-H | pH value | 7.4±0.2 | 7.4±0.1 | 7.4±0.1 | 7.4±0.1 | 7.4±0.1 |
|  | Hydrodynamic diameter (nm) | 114.7  ±0.2 | 113.7  ±1.3 | 113.7  ±1.9 | 112.6  ±0.8 | 112.9  ±1.5 |
|  | PDI | 0.224  ±0.019 | 0.223  ±0.015 | 0.207  ±0.021 | 0.233  ±0.016 | 0.229  ±0.022 |
|  | Zeta potential  (mV) | -58.4  ±2.9 | -53.7  ±3.0 | -45.7  ±2.3 | -43.9  ±5.0 | -53.2  ±5.0 |
| PSLipos-L | pH value | 7.4±0.1 | 7.4±0.1 | 7.3±0.2 | 7.3±0.1 | 7.3±0.2 |
|  | Hydrodynamic diameter (nm) | 128.6  ±0.3 | 127.8  ±2.2 | 126.8  ±3.5 | 127.6  ±1.4 | 127.0  ±0.5 |
|  | PDI | 0.236  ±0.013 | 0.246  ±0.014 | 0.226  ±0.005 | 0.233  ±0.009 | 0.233  ±0.012 |
|  | Zeta potential  (mV) | -68.1  ±1.5 | -71.1  ±4.2 | -59.2  ±4.2 | -54.8  ±4.1 | -61.1  ±9.6 |
| PSLipos-H-NAC | pH value | 7.3±0.1 | 7.3±0.1 | 7.3±0.1 | 7.3±0.1 | 7.2±0.1 |
|  | Hydrodynamic diameter (nm) | 108.9  ±0.7 | 108.3  ±0.8 | 111.3  ±0.9 | 129.6  ±1.1 | 201.8  ±8.1 |
|  | PDI | 0.195  ±0.010 | 0.193  ±0.028 | 0.183  ±0.021 | 0.195  ±0.040 | 0.281  ±0.025 |
|  | Zeta potential  (mV) | -50.3  ±3.9 | -57.7  ±2.8 | -41.4  ±2.7 | -56.9  ±4.4 | -68.9  ±0.4 |
| PSLipos-L-NAC | pH value | 7.3±0.1 | 7.3±0.1 | 7.3±0.1 | 7.3±0.2 | 7.3±0.2 |
|  | Hydrodynamic diameter (nm) | 125.5  ±0.9 | 126.5  ±0.7 | 124.5  ±0.5 | 124.6  ±2.2 | 125.9  ±1.4 |
|  | PDI | 0.255  ±0.011 | 0.211  ±0.026 | 0.235  ±0.017 | 0.215  ±0.006 | 0.235  ±0.026 |
|  | Zeta potential  (mV) | -58.4  ±3.0 | -53.6  ±2.4 | -30.4  ±3.0 | -51.2  ±3.4 | -50.7  ±4.6 |
